# Load-induced increase in muscle activity during 30° abduction in patients with rotator cuff tears and control subjects

**DOI:** 10.1186/s10195-023-00720-8

**Published:** 2023-08-04

**Authors:** Eleonora Croci, Fabian Warmuth, Cornelia Baum, Balazs Krisztian Kovacs, Corina Nüesch, Daniel Baumgartner, Andreas Marc Müller, Annegret Mündermann

**Affiliations:** 1 Department of Biomedical Engineering, University of Basel, Basel, Switzerland; 2 Department of Orthopaedics and Traumatology, University Hospital Basel, Basel, Switzerland; 3 Research and Development, Shoulder and Elbow Surgery, Schulthess Klinik, Zurich, Switzerland; 4 Department of Radiology, University Hospital Basel, Basel, Switzerland; 5 Department of Spine Surgery, University Hospital Basel, Basel, Switzerland; 6 Department of Clinical Research, University of Basel, Basel, Switzerland; 7 IMES Institute of Mechanical Systems, Zurich University of Applied Sciences, Winterthur, Switzerland

**Keywords:** Shoulder, Rotator cuff tear, Rotator cuff tendinopathy, Electromyography, Abduction, Scaption, Motion capture, Muscle activity, Magnetic resonance, Load

## Abstract

**Background:**

Rotator cuff muscles stabilise the glenohumeral joint and contribute to the initial abduction phase with other shoulder muscles. This study aimed to determine if the load-induced increase in shoulder muscle activity during a 30° abduction test is influenced by asymptomatic or symptomatic rotator cuff pathologies.

**Materials and Methods:**

Twenty-five patients with unilateral rotator cuff tears (age, 64.3 ± 10.2 years), 25 older control subjects (55.4 ± 8.2 years) and 25 younger control subjects (26.1 ± 2.3 years) participated in this study. Participants performed a bilateral 30° arm abduction and adduction movement in the scapular plane with handheld weights (0–4 kg). Activity of the deltoid, infraspinatus, biceps brachii, pectoralis major, latissimus dorsi and upper trapezius muscles was analysed at maximum abduction angle after normalisation to maximum voluntary contraction. Shoulders were classified into rotator cuff tendinopathy, asymptomatic and symptomatic rotator cuff tears, and healthy based on magnetic resonance images. A linear mixed model (loads, shoulder types) with random effects (shoulder identification) was applied to the log-transformed muscle activities.

**Results:**

Muscle activity increased with increasing load in all muscles and shoulder types (*P* < 0.001), and 1-kg increments in additional weights were significant (*P* < 0.001). Significant effects of rotator cuff pathologies were found for all muscles analysed (*P* < 0.05). In all muscles, activity was at least 20% higher in symptomatic rotator cuff tears than in healthy shoulders (*P* < 0.001). Symptomatic rotator cuff tears showed 20–32% higher posterior deltoid (*P* < 0.05) and 19–25% higher pectoralis major (*P* < 0.01) activity when compared with asymptomatic tears.

**Conclusions:**

Rotator cuff pathologies are associated with greater relative activity of shoulder muscles, even with low levels of additional load. Therefore, the inclusion of loaded shoulder tests in the diagnosis and rehabilitation of rotator cuff pathologies can provide important insight into the functional status of shoulders and can be used to guide treatment decisions.

*Level of evidence:* Level 2.

*Trial registration*: Ethical approval was obtained from the regional ethics committee (Ethics Committee Northwest Switzerland EKNZ 2021-00182), and the study was registered at clinicaltrials.gov on 29 March 2021 (trial registration number NCT04819724, https://clinicaltrials.gov/ct2/show/NCT04819724).

**Supplementary Information:**

The online version contains supplementary material available at 10.1186/s10195-023-00720-8.

## Introduction

Rotator cuff tears are very common; their prevalence steadily increases with age [1] and approximately 45% of the population over the age of 70 years are affected [2]. Rotator cuff tears can significantly affect shoulder comfort and function during activities of daily living [3], resulting in lower functional scores [4], such as the clinically commonly used Constant score, which takes into account pain, activities of daily living, range of motion and strength [5, 6]. Rotator cuff tears can present clinically in a variety of ways: some patients have severe pain and limited range of motion, while others have no symptoms [7]. Many of these asymptomatic rotator cuff tears eventually become symptomatic within 3 years of initial diagnosis [8, 9].

Rotator cuff muscles primarily facilitate shoulder motion and centre the glenohumeral joint to prevent superior migration of the humeral head [10–12]. Rotator cuff muscles (supraspinatus and infraspinatus), along with other shoulder muscles (deltoid and axioscapular muscles), contribute to the initiation of abduction [13]. For shoulder joint motion to occur smoothly and efficiently, a coordinated force couple of the deltoid and rotator cuff muscles is required, with the infraspinatus, teres minor and subscapularis stabilising the humeral head against the glenoid and providing a fulcrum for the actions of the deltoid and supraspinatus muscle [14]. Rotator cuff tears lead to an imbalance of this force couple and may cause instability of the glenohumeral joint [15]. Although altered kinematics have been reported, a clear consensus has yet to be reached [16, 17]. These kinematic changes may also be associated with altered muscle activity as measured by electromyography (EMG) [18–22].

Changes in muscle activity in rotator cuff pathologies are not completely understood, and the current literature is inconsistent. For instance, a tendency to increased shoulder muscle activity has been found in patients with rotator cuff tears compared with normal subjects [18]. Increased axioscapular muscle activity has also been found to be a compensatory mechanisms for the destabilising forces of the deltoid muscle [21], suggesting decreased glenohumeral joint motion [23]. However, symptomatic rotator cuff tears have less activity of the deltoid muscle during shoulder elevation compared with asymptomatic tears [23]. Moreover, muscle activity of only the posterior deltoid and biceps brachii was higher in symptomatic rotator cuff tears than in age-matched healthy controls [19].

To date, the differences in muscle activity between symptomatic and asymptomatic rotator cuff tears have not been fully elucidated [23]. In addition, the effect of additional load during a dynamic task in rotator cuff tears has not yet been fully studied even in control subjects [24, 25]. Previous studies have shown that deltoid and rotator cuff muscle activity in asymptomatic shoulders increased with additional load during the first 90° of abduction in the scapular plane [24], and that increasing load in asymptomatic shoulders resulted in higher activation of the middle deltoid, rotator cuff and axioscapular muscles throughout abduction in the scapular plane [25]. Understanding muscle activation patterns under different loading conditions is important for rehabilitation because increased loading is commonly used in shoulder rehabilitation programmes to gradually challenge muscles to improve muscle strength and function [24, 26].

This study aimed to investigate whether the load-induced increase in shoulder muscle activity during 30° arm abduction in the scapular plane is influenced by asymptomatic or symptomatic pathologies of the rotator cuff. We hypothesised that muscle activity would increase with increasing loading conditions and that the load-induced increase in deltoid muscle activity would be greater in the presence of a rotator cuff pathology to compensate for rotator cuff deficiency.

## Materials and methods

### Participants

In this study, 25 patients with unilateral rotator cuff tears, 25 older (45–85 years) and sex-matched control subjects, and 25 younger (20–30 years) and sex-matched control subjects were recruited from our clinic (patients) or from the surrounding community via advertisements (control subjects) between May 2021 and January 2023. Two controls groups were chosen to include both shoulders with asymptomatic rotator cuff tears (to be expected in the older control group) and healthy shoulders. Inclusion criteria for patients were: age between 45 and 85 years, a unilateral rotator cuff tear with at least the supraspinatus tendon affected (either a partial or a complete tear) confirmed by diagnostic imaging, an active arm range of motion of at least 30° in abduction and flexion, and no known clinical history or pain in the contralateral glenohumeral joint. Control subjects were included if they were aged between 45 and 85 years or between 20 and 30 years, had an active arm range of motion of at least 90° in abduction and flexion, and had no history of injury or pain in both shoulders. General exclusion criteria were: body mass index (BMI) greater than 35 kg/m^2^, previous surgical treatment of the upper extremities, neuromuscular disorders affecting upper limb motion, and other pathologies influencing shoulder joint mobility. Written informed consent was obtained before data collection. This study was part of a larger umbrella study [27] approved by the regional ethics board and conducted in accordance with the Declaration of Helsinki (2013) [28]. As described in detail in [27], for a 5% significance level, 90% power, and a correlation of 0.37, a sample size of 76 shoulders was necessary.

### Experimental protocol

**Fig. 1:**
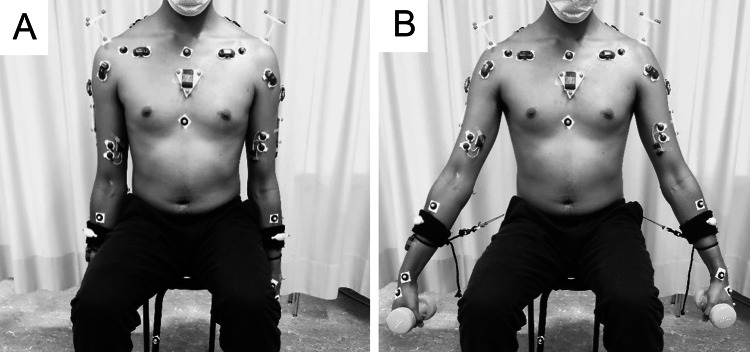
Setting of the 30° abduction test at minimum (**A**) and maximum (**B**) range of motion. Participants performed a sequence of three abduction–adduction repetitions in the scapular plane. The second repetition was included in the analysis

First, the Constant score [5, 6] was obtained for both shoulders of all participants to determine the overall status of their shoulders. Then, all participants performed a sequence of three repetitions of a 30° arm abduction and adduction movement in the scapular plane with elbows extended and hands in neutral positions, with and without additional handheld weights (0, 1, 2, 3 and 4 kg). This test was performed simultaneously with both arms to ensure a straight and stable torso, first without handheld weights and then in random order with the handheld weights. The repetitions were interspersed with 1-min rests. To limit the range of motion of the task to 30°, adjustable strings were attached to the hip and forearms. The length of the strings was assessed with a goniometer (Fig. 1). Verbal commands were given to the participants to maintain a similar movement velocity, which corresponded to about 4.5 s for a 30° abduction and adduction movement. Testing was performed while motion capture (sampling rate of 240 Hz, Vicon, Oxford, UK) and EMG data (sampling rate of 2400 Hz, Myon AG, Schwarzenberg, Switzerland) were acquired. Subjects were equipped with 53 reflective anatomical and cluster markers and 16 EMG sensors [27]. The reflective markers were placed on landmarks of the upper extremities and torso according to the International Society of Biomechanics guidelines [29]. After skin preparation, the EMG sensors were placed parallel to the muscle fibres according to the surface EMG for the non-invasive assessment of muscles guidelines (SENIAM) [30] and Criswell [31]. Muscle activities of the deltoid (anterior, middle and posterior part), infraspinatus, biceps brachii, latissimus dorsi, pectoralis major and upper trapezius muscles of both sides were recorded.

In addition, muscle activities of maximum voluntary isometric contractions (MVCs) were recorded. The following isometric tests were performed: empty can test, internal rotation (at 90° shoulder abduction and 90° elbow flexion), shoulder flexion at 125°, palm press (at 90° shoulder flexion and 20° elbow flexion) and shoulder extension (at 30° abduction with internal rotation) [32, 33]. Elbow flexion at 90° was also performed to obtain the MVC of the biceps brachii muscle. Each contraction was performed once for 5 s.

Magnetic resonance images (MRI) of all shoulders (of all patients and controls) were obtained using a 3-T scanner (Prisma, Siemens Healthineers, Erlangen, Germany) with dedicated shoulder and body array coils. No contrast agent was administered. The following sequences were acquired: axial proton density turbo spin echo sequence with fat saturation, parasagittal T1-weighted turbo spin echo, parasagittal and coronal T2-weighted BLADE and coronal T1-weighted volumetric interpolated breath-hold examination Dixon sequence.

### Data processing

According to MRI findings, participants were grouped into the following shoulder types: healthy, with rotator cuff tendinopathy, with asymptomatic rotator cuff tears and with symptomatic rotator cuff tears (confirming the initial diagnosis). If any other findings were present, but none on the rotator cuff, data of the corresponding shoulders were excluded from the analyses. An experienced radiologist in musculoskeletal imaging read all MRIs.

All data were processed using MATLAB 2021b (MathWorks Inc., Natick). EMG data were filtered with a fourth-order bandpass filter (10–450 Hz) [34], except for the latissimus dorsi and pectoralis major, where a modified bandpass filter (40–450 Hz) was used to reduce electrocardiographic artefacts. EMG data were then full-wave rectified and smoothed with a 100-ms moving average window. The EMG signals of the abduction tests were then normalised to the maximum values of the MVCs for each muscle. The mean intensity of the interval 250 ms before to 250 ms after the maximum abduction angle of the second repetition was computed and used for further analysis. Kinematics data were reconstructed, and glenohumeral joint centres were estimated using the regression equation published by the International Shoulder Group [35]. Abduction angles were calculated using the XZY sequence [36].

### Statistical analysis

All statistical analyses were performed using R statistical software [37]. All data were checked for normality using Shapiro–Wilk’s test, and log transformation, parametric and nonparametric tests were applied as appropriate. Differences in the Constant score between shoulder groups were evaluated using univariate analysis of variance with independent *t*-test post hoc tests with Bonferroni correction. Muscle activities were log transformed [log_10_(EMG + 1)] to achieve normality. Linear mixed models [38] with fixed (loads and shoulder types) and random effects (shoulder identification, to account for intra- and inter-individual differences within and between subjects) were applied to the log-transformed activities of each muscle with healthy shoulders as reference. Post hoc tests (estimated marginal means [39]) were carried out accordingly. The level of significance was set a priori to 5%.

## Results

Overall, 25 patients with unilateral rotator cuff tear (15 men, 10 women; mean (standard deviation) age: 64.3 (10.2) years, height: 172 (10) cm, body mass: 78.4 (17.3) kg, BMI: 26.5 (5.0) kg/m^2^); 25 older control subjects (15 men, 10 women, age: 55.4 (8.2) years, height: 174 (9) cm, body mass: 76.5 (13.0) kg, BMI: 25.2 (4.6) kg/m^2^); and 25 younger control subjects (15 men, 10 women, age: 26.1 (2.3) years, height: 177 (9) cm, body mass: 71.6 (12.9) kg, BMI: 22.7 (3.0) kg/m^2^) participated in this study.

**Fig. 2:**
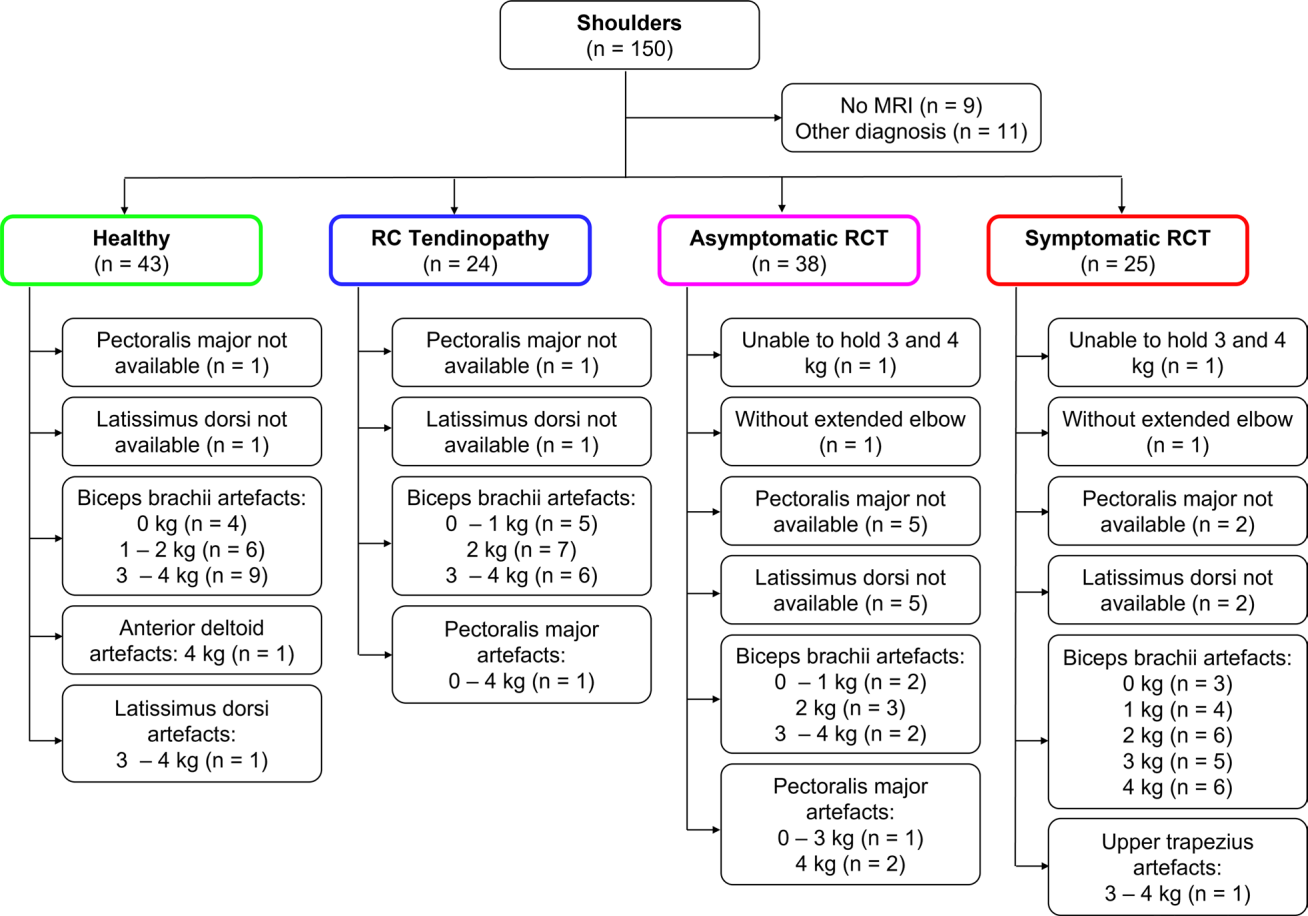
Flowchart illustrating the classification of all included shoulders into different shoulder types. *RC* rotator cuff, *RCT* rotator cuff tear, *MRI* magnetic resonance imaging

Because of missing MRIs or other diagnosis not involving the rotator cuff, 20 shoulders (5 contralateral shoulders of patients, 10 shoulders of older control subjects and 5 shoulders of younger control subjects) were excluded from the analysis (Fig. 2). MRI findings resulted in the following shoulder groups included in the analysis: 43 healthy, 24 with rotator cuff tendinopathy, 38 with asymptomatic rotator cuff tears, and 25 with symptomatic rotator cuff tears (Fig. 2). It should be noted that the healthy shoulders were predominantly from the younger control subjects (83.7%). All subjects were able to perform the loaded and unloaded abduction tests with both arms, except for two patients who were unable to hold 3 kg and 4 kg or perform the test correctly with elbows extended. In addition, EMG data of the pectoralis major and latissimus dorsi muscles were not available for nine shoulders, and some other EMG data had to be excluded due to signal artefacts (Fig. 2, Additional File 1).

### Constant score

Constant scores of shoulders with symptomatic rotator cuff tears were lower than those of all other shoulder types [*P* < 0.001; mean (standard deviation), shoulders with rotator cuff tendinopathy: 85.2 (6.1); shoulders with asymptomatic rotator cuff tears: 84.0 (5.2); shoulders with symptomatic rotator cuff tear: 74.0 (10.3); healthy shoulders: 88.2 (2.6)]. Constant scores of shoulders with asymptomatic rotator cuff tears were also lower than those of healthy shoulders (*P* < 0.001). Constant scores did not differ between asymptomatic rotator cuff tears and shoulders with rotator cuff tendinopathy (*P* = 1.000) or between shoulders with rotator cuff tendinopathy and healthy shoulders (*P* = 0.187). Shoulders with symptomatic rotator cuff tears had some pain and range of motion deficit compared with the other shoulder types. The other differences were due to the different scores achieved in the strength component of the Constant score.

### Overall effect of shoulder type and load on muscle activity

**Fig. 3:**
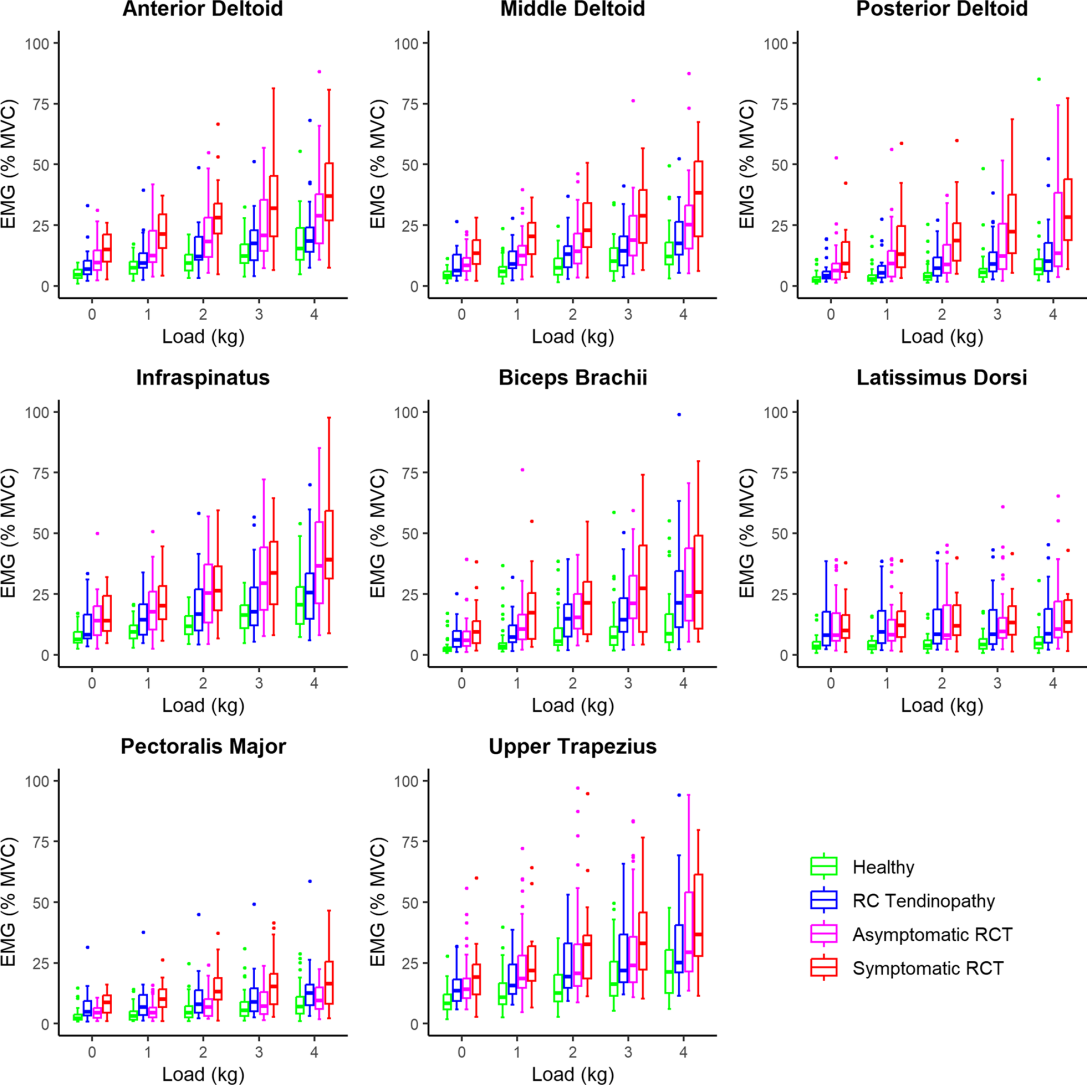
Box plots of the normalised muscle activities. *RC* rotator cuff, *RCT* rotator cuff tear, *MVC* maximal voluntary contraction, *EMG* electromyography

**Fig. 4:**
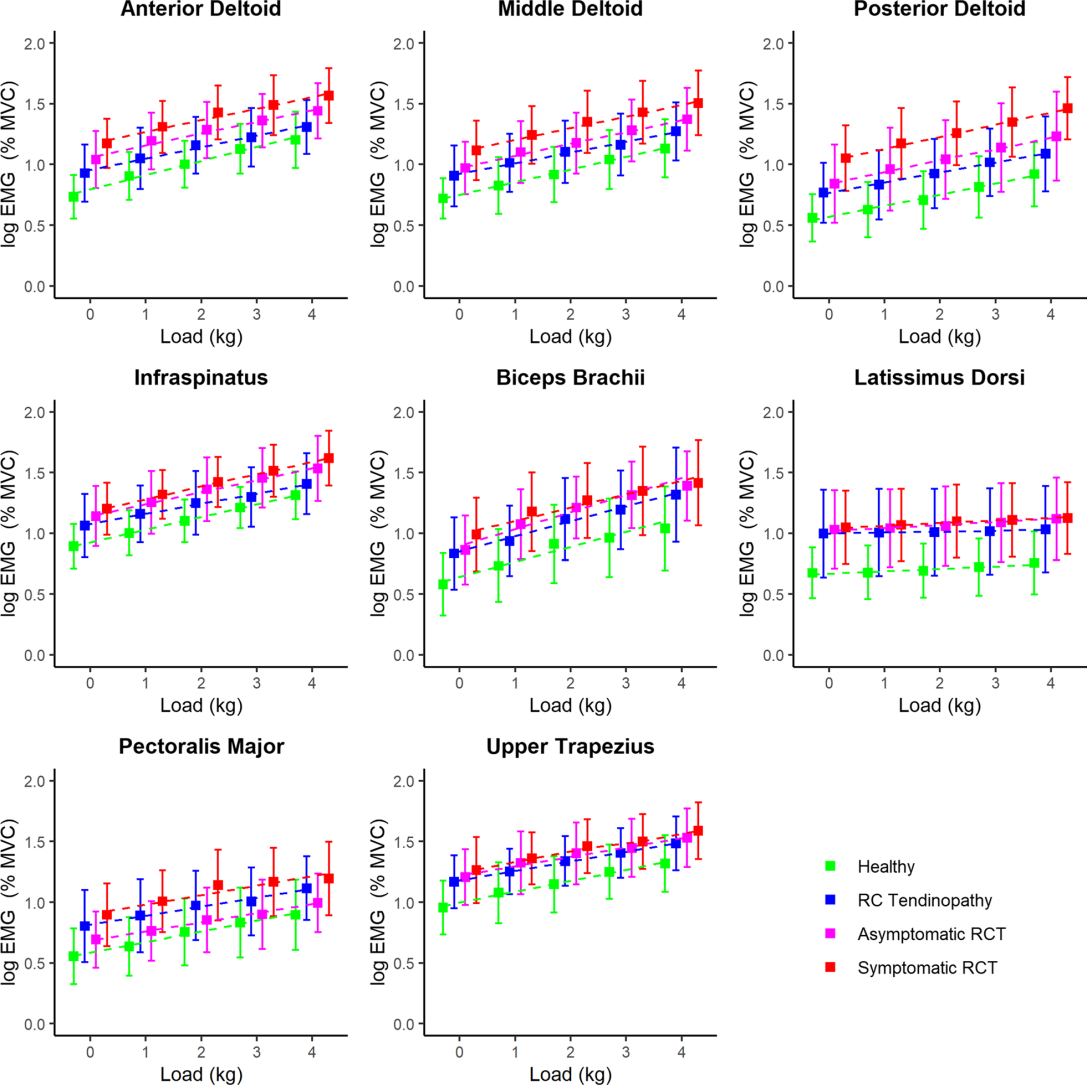
Mean and standard deviation of the normalised and log-transformed muscle activities with regression lines. *RC* rotator cuff, *RCT* rotator cuff tear, *MVC* maximal voluntary contraction, *EMG* electromyography

The following pattern in MVC-normalised muscle activity was observed in all loading conditions: lowest in healthy subjects, slightly higher in rotator cuff tendinopathy, then substantially higher in asymptomatic rotator cuff tears and finally highest in symptomatic rotator cuff tears (Fig. 3). This trend was observed in all muscles except for the pectoralis major, where shoulders with rotator cuff tendinopathy had higher muscle activity than shoulders with asymptomatic rotator cuff tears (Fig. 4, Table 1). In almost all muscles studied, muscle activity without additional weight was as high in symptomatic rotor cuff tears as in healthy shoulders with 4 kg additional weight (Fig. 3, median and interquartile range in Additional file 2). Particularly, the latissimus dorsi muscle activity without additional weight in symptomatic rotator cuff tears was twice as high as muscle activity with 4 kg additional weight in healthy subjects (Fig. 3, Additional file 2). In addition, a significant increase in muscle activity with increasing load was observed for all muscles and shoulder types (Figs. 3 and 4, Table 1). The estimated coefficients for load were similar for all muscles except for the latissimus dorsi muscle, where muscle activity increased only slightly with increasing load (Fig. 4, Table 1). Results (*P*-values) of the linear mixed models with the different shoulders as reference can be found in the Additional file 3.

**Table 1:** Effects load and shoulder types of linear mixed models on the log-transformed muscle activities

Predictors	Anterior deltoid	Middle deltoid	Posterior deltoid	Infraspinatus	Biceps brachii	Latissimus dorsi	Pectoralis major	Upper trapezius
Intercept								
Estimates	0.76	0.72	0.54	0.90	0.61	0.66	0.56	0.97
CI	[0.70–0.83]	[0.65–0.79]	[0.46–0.63]	[0.83–0.96]	[0.51–0.70]	[0.58–0.75]	[0.48–0.64]	[0.90–1.04]
*P*-values	** < 0.001**	** < 0.001**	** < 0.001**	** < 0.001**	** < 0.001**	** < 0.001**	** < 0.001**	** < 0.001**
Load								
Estimates	0.12	0.10	0.09	0.10	0.12	0.02	0.09	0.09
CI	[0.11 to 0.12]	[0.10 to 0.11]	[0.08 to 0.36]	[0.09 to 0.11]	[0.11 to 0.14]	[0.01 to 0.02]	[0.08 to 0.10]	[0.08 to 0.10]
*P*	** < 0.001**	** < 0.001**	** < 0.001**	** < 0.001**	** < 0.001**	** < 0.001**	** < 0.001**	** < 0.001**
RC tendinopathy								
Estimates	0.19	0.20	0.22	0.17	0.24	0.33	0.25	0.20
CI	[0.08 to 0.29]	[0.08 to 0.31]	[0.08 to 0.36]	[0.07 to 0.28]	[0.07 to 0.40]	[0.18 to 0.48]	[0.12 to 0.38]	[0.09 to 0.32]
*P*-values	** < 0.001**	**0.001**	**0.002**	**0.002**	**0.006**	** < 0.001**	** < 0.001**	**0.001**
Asymptomatic RCT								
Estimates	0.31	0.26	0.31	0.26	0.30	0.36	0.13	0.26
CI	[0.21 to 0.40]	[0.16 to 0.37]	[0.18 to 0.43]	[0.16 to 0.35]	[0.16 to 0.44]	[0.22 to 0.49]	[0.01 to 0.25]	[0.16 to 0.36]
*P*-values	** < 0.001**	** < 0.001**	** < 0.001**	** < 0.001**	** < 0.001**	** < 0.001**	**0.031**	** < 0.001**
Symptomatic RCT								
Estimates	0.44	0.41	0.52	0.31	0.42	0.38	0.37	0.30
CI	[0.33 to 0.54]	[0.30 to 0.53]	[0.38 to 0.66]	[0.20 to 0.42]	[0.26 to 0.58]	[0.23 to 0.54]	[0.23 to 0.50]	[0.19 to 0.41]
*P*-values	** < 0.001**	** < 0.001**	** < 0.001**	** < 0.001**	** < 0.001**	** < 0.001**	** < 0.001**	** < 0.001**
Load × RC tendinopathy								
Estimates	−0.02	−0.02	−0.01	−0.02	−0.00	−0.01	−0.01	−0.01
CI	[−0.04 to −0.01]	[−0.03 to 0.00]	[−0.02 to 0.01]	[−0.03 to −0.00]	[−0.02 to 0.02]	[−0.02 to −0.00]	[−0.03 to 0.00]	[−0.02 to 0.00]
*P*-values	**< 0.001**	**0.011**	0.252	**< 0.001**	0.985	**0.011**	0.135	0.068
Load × asymptomatic RCT								
Estimates	−0.02	0.01	0.01	−0.00	0.02	0.01	−0.01	−0.01
CI	[−0.03 to −0.01]	[−0.01 to 0.01]	[−0.01 to 0.02]	[−0.01 to 0.01]	[−0.00 to 0.04]	[−0.00 to 0.01]	[−0.03 to 0.00]	[−0.02 to −0.00]
*P*-values	**0.003**	0.600	0.266	0.447	0.060	0.171	0.139	**0.027**
Load × symptomatic RCT								
Estimates	−0.02	−0.01	0.01	0.00	−0.01	0.00	−0.01	−0.00
CI	[−0.03 to −0.01]	[−0.02 to −0.01]	[−0.02 to 0.02]	[−0.01 to 0.01]	[−0.03 to 0.01]	[−0.01 to 0.01]	[−0.03 to 0.01]	[−0.02 to 0.01]
*P*-values	**0.006**	0.350	0.317	0.759	0.237	0.571	0.291	0.633

Bold values indicate significant differences (*P* < 0.05)

*RC* rotator cuff, *RCT* rotator cuff tear, *CI* confidence interval

### Interaction effect of load magnitude and shoulder type

Few interaction effects of the linear mixed model were found in the anterior deltoid muscle (load with rotator cuff tendinopathy, with asymptomatic rotator cuff tears, and with symptomatic rotator cuff tears), middle deltoid muscle (load with rotator cuff tendinopathy), infraspinatus muscle (load with rotator cuff tendinopathy), latissimus dorsi muscle (load with rotator cuff tendinopathy), and upper trapezius muscle (load with asymptomatic rotator cuff tears, Table 1). In these cases, the load-induced increase in muscle activity was less steep than in healthy shoulders (Fig. 4, Table 1).

### Effect of individual shoulder

In all models of muscle activity, fixed effects (marginal R^2^) explained less variance than random effects (conditional R^2^), especially in the case of latissimus dorsi (Table 2). High intraclass correlation coefficients were found in all models, so most of the variance was explained by the random factor (Table 2).

**Table 2:** Random effects of the linear mixed models for the log-transformed muscle activities

Muscle	ICC	Marginal R^2^	Conditional R^2^
Anterior deltoid	0.865	0.475	0.929
Middle deltoid	0.904	0.416	0.944
Posterior deltoid	0.897	0.400	0.938
Infraspinatus	0.889	0.412	0.935
Biceps brachii	0.858	0.370	0.911
Latissimus dorsi	0.967	0.258	0.976
Pectoralis major	0.833	0.288	0.881
Upper trapezius	0.892	0.335	0.928

*ICC* intraclass correlation coefficient

### Effect of load on muscle activity

Post hoc tests for the load effect showed that the increase in muscle activity was significant from a load increment of 1 kg in all muscles and all shoulder types (*P* < 0.001 for all). The only exception was latissimus dorsi muscle activity in shoulders with rotator cuff tendinopathy (*P* = 0.153 for all loads compared).

### Effect of shoulder type on muscle activity at each load

Results of the post hoc test for shoulder effects are shown in Additional file 4, and only statistically significant results are reported here. Symptomatic rotator cuff tears showed higher muscle activity than healthy shoulders in all analysed muscles (at all loads). The increase in the log-transformed muscle activity was greatest in the posterior deltoid (ranging from 58% to 88%) and least in the upper trapezius (ranging from 20 to 32%). Similarly, asymptomatic rotator cuff tears had higher muscle activity than healthy shoulders in all muscles (at all loads), but not in the pectoralis major (at all loads). Indeed, pectoralis major muscle activity was higher in symptomatic rotator cuff tears than in asymptomatic tears at all loading conditions, with increases ranging from 20% to 32%. Differences between symptomatic and asymptomatic rotator cuff tears were also found in posterior deltoid muscle activity at all loads (between 19% and 25% higher activity in symptomatic rotator cuff tears). Higher muscle activity in shoulders with rotator cuff tendinopathy than healthy shoulders was found in the biceps brachii (22% to 44% increase), latissimus dorsi (37% to 48% increase) and pectoralis major (21 to 44% increase) at all loads, but in the infraspinatus and deltoid muscles only at some loads. These were in the infraspinatus at 0 kg and 1 kg (15% to 16% increase), in the anterior deltoid at 0 kg and 1 kg (16% to 26% increase), in the middle deltoid at 0, 1, and 2 kg (21% to 26% increase), and in the posterior deltoid at all loads but 4 kg (25% to 37% increase). The only difference between shoulders with asymptomatic rotator cuff tears and shoulders with rotator cuff tendinopathy was higher infraspinatus muscle activity with 4-kg additional weight (9%) in shoulders with asymptomatic rotator cuff tears. In contrast, shoulders with symptomatic rotator cuff tears had higher muscle activity in the three parts of the deltoid muscle at all loads (20% to 41% increase) and also in the infraspinatus muscle at loads of at least 2 kg (14% to 16% increase) compared with shoulders with rotator cuff tendinopathy.

## Discussion

In this study, muscle activity of 130 shoulders was analysed during a 30° arm abduction and adduction movement with and without additional handheld weight. Although only 25 patients with unilateral rotator cuff tears were recruited, incidental MRI findings were discovered in 62 additional shoulders. These results allowed a realistic evaluation of the load-induced increase in activity in shoulder muscles in different rotator cuff pathologies.

### Load-induced increase in muscle activity

In our abduction test, a load-induced increase in muscle activity was observed in all shoulder muscles and types studied, where every 1-kg load increment resulted in a significant increase in muscle activity. The only exception was the activity of the latissimus dorsi muscle in shoulders with rotator cuff tendinopathy, which was less affected by additional weights. This low activation of latissimus dorsi at maximum abduction angle could be explained by its function, which is mainly arm adduction and internal rotation [12]. A load-induced increase in deltoid and rotator cuff muscle activity was also observed by Alpert et al. [24] in healthy subjects during the first 90° of abduction in the scapular plane. Analogous to that study, we observed similar muscle activity of the anterior and middle deltoid and a slightly lower muscle activity of the posterior deltoid. This is related to the individual muscle force vectors and muscle moment arms of the three parts of the deltoid. Indeed, the posterior deltoid muscle has a short lever arm during the first phase of full abduction [40]; moreover, the loaded and unloaded test was performed with the arm in neutral rotation, so no increased muscle activity of the posterior deltoid was required. The load-induced increase in muscle activity of the major muscles producing abduction torque (deltoid) is required in healthy subjects during the first 90° of abduction in the scapular plane to execute the movement. A systematic increase with additional weights in upper trapezius muscle activity during arm abduction in the scapular plane was also observed in asymptomatic subjects by Reed et al. [25]. This load-induced increase in activity of the other shoulder muscles is biomechanically supported because greater activity of the deltoid muscle causes potential translational forces on the humerus [14, 40], possibly leading to subacromial impingement, such that the rotator cuff and axioscapular muscles require increased muscle activity to counterbalance these forces [41]. We also observed an increase in muscle activity of the pectoralis major and biceps brachii muscles, possibly to oppose the action of the deltoid muscle, hence acting as a humeral head depressor. With increasing load, we also noted increased variability in muscle activity, which could be due to the individual strength capacity of the participants, as the additional weights were not scaled to the relative maximum strength. However, even in this case, the variability would have tended to increase with increasing load [24, 25].

### Load-induced changes after rotator cuff pathologies

Overall, shoulders with rotator cuff tears or tendinopathy showed the same load-induced increase in muscle activity as healthy shoulders, but relative muscle activity was higher in these patients. Moreover, symptomatic rotator cuff tears had higher muscle activity than asymptomatic rotator cuff tears, and these in turn had higher muscle activity than rotator cuff tendinopathies. In the latter case, there was one exception: pectoralis major muscle activity was lower in asymptomatic rotator cuff tears than in rotator cuff tendinopathy. This higher muscle activity in shoulders with rotator cuff pathology is consistent with the study of Kelly et al. [18], which concluded that patients with rotator cuff tears tend to have higher muscle activity compared with normal subjects, regardless of the presence of pain or symptoms. However, in contrast to the study of Shinozaki et al. [23], which used positron emission tomography (PET), we found no decrease in deltoid muscle activity and no increase in trapezius muscle activity in symptomatic rotator cuff tears compared with asymptomatic rotator cuff tears. This is likely due to the different methodologies and acquisition time of PET and EMG, and differences in the movement performed (arm in internal rotation versus neutral rotation). Although differences in upper trapezius muscle activity between symptomatic and asymptomatic rotator cuffs were not significant (log-transformed data), upper trapezius muscle activity tended to be higher, suggesting less glenohumeral joint motion in symptomatic rotator cuff tears. Significant differences between symptomatic and asymptomatic rotator cuff tears were found only in posterior deltoid and pectoralis major muscle activity, where the activity was greater in the symptomatic shoulders. One possible explanation may be that tears are more severe in symptomatic shoulders, leading to even greater compensation of the greater deltoid for the deficient rotator cuff and higher pectoralis muscle activity to counterbalance the superior translational forces of the deltoid muscle.

In another study, patients with symptomatic rotator cuff tears were found to have higher posterior deltoid and biceps brachii muscle activity, especially during weight lifting, compared with age-matched healthy controls [19]. Consequently, this study supported the benefit of treating the long head of the biceps tendon in symptomatic rotator cuff tears, as the biceps brachii can act as humeral depressor and cause pain if over-activated. This was only partially confirmed by our results, because in symptomatic rotator cuff tears, muscle activity of the biceps brachii and posterior deltoid was increased compared with healthy shoulders, but also the activity of the other muscles studied was increased. The increase in muscle activity was highest in the posterior deltoid muscle. While in healthy shoulders the muscle activity of the posterior deltoid is much lower than that of the anterior and middle deltoids, in shoulders with rotator cuff tears the posterior deltoid is rather active and its activity level approaches that of the anterior and middle deltoids. Therefore, the posterior deltoid muscle gains importance in abduction movement up to 30° in the scapular plane after rotator cuff tears. The observed increase in latissimus dorsi muscle activity is consistent with the study by Hawkes et al. [21]. Indeed, an increase in muscle activity of the biceps brachii, upper trapezius–serratus anterior, latissimus dorsi and teres major muscles was observed in massive rotator cuff tears compared with healthy subjects [21], as a compensatory mechanism for the destabilising forces of the deltoid. Similar to symptomatic rotator cuff tears, activity differences in almost all muscles were found between asymptomatic rotator cuff tears and healthy shoulders, but not in the pectoralis major muscle activity.

Clinically, rotator cuff tendinopathy is not as relevant as a rotator cuff tear, yet athletes with rotator cuff tendinopathy had an abnormal pattern of scapular movement that may be related to scapular muscle deficits [42]. During the loaded and unloaded abduction test in our study, shoulders with rotator cuff tendinopathy had higher activity than healthy shoulders in almost all muscles studied at all loads, with the exception of the infraspinatus and deltoid muscles at only some loads. Although latissimus dorsi muscle activity was higher in shoulders with rotator cuff tendinopathy, it remained unchanged with additional load, and deltoid and infraspinatus muscles had reduced activity. It is possible that these changes occur to avoid an overactivation of the tendinopathic supraspinatus muscle. Although the changes in muscle activity in shoulders with rotator cuff tendinopathy may not be as pronounced as in rotator cuff tears, compensatory mechanisms for the pathologic rotator cuff still appears to occur. Understanding the adaptive changes in muscle activity is crucial for rehabilitation as shoulder and scapula muscle activity may be altered with specific interventions such as mobilisation and strengthening exercises [43–44].

### Clinical relevance

The possibility of comparing muscle activity of shoulders with rotator cuff pathologies and healthy shoulders with a simple abduction test is useful in the clinic to gain a better understanding of compensation mechanisms. In this 30° arm abduction test in the scapular plane, not only the deltoid and infraspinatus muscle showed a significant increase in activity with additional loading in all shoulder types, but also the surrounding stabilising muscles. In the Constant score, symptomatic rotator cuff tears differed from all other shoulder types and differences between asymptomatic rotator cuff tears and healthy shoulders were also detected. These differences were also observed in the muscle activity in our 30° arm abduction test. In addition, differences in the muscle activity between shoulders with rotator cuff tendinopathy and shoulders with asymptomatic rotator cuff tears or healthy shoulders were also present. This low abduction angle allows even patients with a limited range of motion to perform this test, and objective measurements of these patients can be obtained. However, performing tests with the same absolute handheld weights in all participants leads to variability due to individual strength capacity that must be taken into account when interpreting results. It is possible that weaker participants require a higher level of muscle activity (closer to maximal contraction to complete the task) or cannot perform the test at all, while stronger participants might not be as challenged by the same handheld weight. The EMG system is portable and the test can be performed by a trained person in less than 15 min, making an implementation in clinical practice feasible.

### Strengths and limitations

A major strength of our study is the combination of EMG data and MRI to investigate differences in muscle activity and potential compensatory mechanisms in shoulders with rotator cuff pathologies. EMG surface electrodes were used in this study, and because of the compact anatomy of the shoulder, the possibility of crosstalk of EMG signals from adjacent muscles cannot be excluded. To compare muscle activity between participants, we normalised the EMG with MVC. However, MVC might be influenced by pain and result in a larger normalised value due to a smaller denominator [46, 47], making it challenging to compare symptomatic (painful) and pain-free shoulders [20]. Alternatively, the amplitude could have been normalised to the unloaded condition of each shoulder, but this would not have excluded evasive muscle activity a priori. We chose the MVC normalisation method because there were no obvious differences in millivolt values between patients’ shoulders compared with the controls (Additional file 5).

Muscle activity exhibits some variability, to which several factors may have contributed. Although verbal instructions were given to the participants to maintain a comparable movement velocity, there could have been variations in movement duration. In addition, movement in the scapular plane was not restricted, and hence slight deviation from the scapular plane may have occurred. Some of the variability in muscle activity may also be explained by the heterogeneity of rotator cuff tears in the participants. However, to further characterise muscle activity for a specific tear type and severity, a larger number of participants would be needed.

Nonetheless, the results of this study are clinically relevant: this 30° abduction test was implemented to investigate the effect of additional handheld weight in shoulders with rotator cuff pathologies, and we indeed observed important biomechanical changes in shoulders with rotator cuff pathologies, such as greater relative activation of shoulder muscles even with small additional load.

## Conclusion

Rotator cuff pathologies are associated with greater relative shoulder muscle activity already at low additional load. In addition to questionnaires and clinical tests, objective measures, such as muscle activity, can be used to better distinguish between pathologies and identify patient-specific deficits and compensation strategies. Incorporating this 30° loaded and unloaded shoulder abduction test in the scapular plane into the diagnosis and rehabilitation of rotator cuff tears may provide important insight into the functional shoulder status and may be used to guide treatment. Handheld weights could be adjusted according to the individual muscle strength capacity for optimal within and between subject comparisons.

### Supplementary Information

**Additional file 1: Table S1.** Number of data points entered in linear mixed models for each muscle, shoulder type and handheld weight.

**Additional file 2: Table S2.** Median and interquartile range of the normalised muscle activity.

**Additional file 3: Table S3.** *P*-values for the effects of load and shoulder types of the linear mixed models on the log-transformed muscle activities with change of reference.

**Additional file 4: Table S3.** *P*-values of the post hoc tests of the log-transformed muscle activities for the shoulder effect.

**Additional file 5: Figure S1.** Absolute difference in the muscle activity of maximum voluntary contraction between sides for each group.

## Data Availability

All data generated or analysed during this study are included in this published article and its supplementary information files.

## Abbreviations

EMG: Electromyography
ICC: Intraclass correlation coefficient
MRI: Magnetic resonance imaging
ms: Milliseconds
mV: Millivolts
MVC: Maximum voluntary isometric contraction
PET: Positron emission tomography
SENIAM: Surface EMG for the non-invasive assessment of muscles guidelines
