# Supplementary material for: Load-induced increase in muscle activity during 30° abduction in patients with rotator cuff tears and control subjects
Source: J Orthop Traumatol. 2023 Aug 4;24:41. doi: 10.1186/s10195-023-00720-8 (PMC10403481; doi:10.1186/s10195-023-00720-8)
Supplement: Supplementary file 1 — Additional file 1: Table S1. Number of data points entered in linear mixed models for each muscle, shoulder type and handheld weight. [file 10195_2023_720_MOESM1_ESM.pdf]

Table S1: Number of data point entered in linear mixed models for each muscle, shoulder type and handheld weight.

**Muscle**	**Healthy**					**RC Tendinopathy**					**Asymptomatic RCT**					**Symptomatic RCT**				
	0kg	*1kg*	*2kg*	*3kg*	*4kg*	*0kg*	*1kg*	*2kg*	*3kg*	*4kg*	*0kg*	*1kg*	*2kg*	*3kg*	*4kg*	*0kg*	*1kg*	*2kg*	*3kg*	*4kg*
Anterior Deltoid	43	43	43	43	42	24	24	24	24	24	37	37	37	36	36	24	24	24	23	23
Middle Deltoid	43	43	43	43	43	24	24	24	24	24	37	37	37	36	36	24	24	24	23	23
Posterior Deltoid	43	43	43	43	43	24	24	24	24	24	37	37	37	36	36	24	24	24	23	23
Infraspinatus	43	43	43	43	43	24	24	24	24	24	37	37	37	36	36	24	24	24	23	23
Biceps Brachii	39	37	37	34	34	19	19	17	18	18	35	35	34	34	34	21	20	18	18	17
Latissimus Dorsi	42	42	42	41	41	23	23	23	23	23	32	32	32	31	31	22	22	22	21	21
Pectoralis Major	42	42	42	42	42	24	24	24	24	24	31	31	31	30	29	22	22	22	21	21
Upper Trapezius	43	43	43	43	43	22	22	22	22	22	37	37	37	36	36	24	24	24	22	22

RC, Rotator cuff; RCT, Rotator cuff tear;
