# Supplementary material for: Load-induced increase in muscle activity during 30° abduction in patients with rotator cuff tears and control subjects
Source: J Orthop Traumatol. 2023 Aug 4;24:41. doi: 10.1186/s10195-023-00720-8 (PMC10403481; doi:10.1186/s10195-023-00720-8)
Supplement: Supplementary file 2 — Additional file 2: Table S2. Median and interquartile range of the normalised muscle activity. [file 10195_2023_720_MOESM2_ESM.pdf]

*Table S2*: Median and interquartile range of the normalized muscle activity.

| | | **Muscle activities (%MVC)** Median (interquartile range) | | | | |
|---|---|---|---|---|---|---|
| | | *0kg* | *1kg* | *2kg* | *3kg* | *4kg* |
| **Anterior Deltoid** | | | | | | |
| | Healthy | 4.5 (3.1–6.6) | 7.5 (5.0–9.9) | 9.4 (6.3–12.8) | 12.3 (9.4–17.1) | 15.4 (10.8–23.8) |
| | RC Tendinopathy | 6.8 (4.8–10.4) | 9.3 (7.4–13.5) | 12.1 (10.8–20.3) | 17.5 (10.6–22.9) | 18.5 (14.1–24.0) |
| | Asymptomatic RCT | 9.6 (6.4–14.5) | 12.5 (9.7–22.6) | 18.2 (11.9–28.0) | 20.8 (14.4–35.4) | 28.9 (17.4–37.8) |
| | Symptomatic RCT | 15.0 (9.9–21.1) | 21.4 (15.6–29.4) | 28.1 (21.5–33.9) | 32.0 (20.4–45.2) | 37.0 (26.9–50.4) |
| **Middle Deltoid** | | | | | | |
| | Healthy | 4.2 (3.1–6.0) | 5.9 (3.7–7.8) | 7.4 (4.6–11.3) | 10.1 (6.1–15.6) | 12.1 (8.8–17.9) |
| | RC Tendinopathy | 6.3 (4.2–12.9) | 8.9 (7.3–14.1) | 13.1 (7.6–16.4) | 14.3 (8.5–20.3) | 17.4 (12.9–26.4) |
| | Asymptomatic RCT | 8.4 (6.1–11.5) | 12.4 (8.7–16.5) | 14.2 (9.1–21.6) | 18.8 (12.4–28.8) | 25.2 (15.4–33.1) |
| | Symptomatic RCT | 13.4 (9.0–18.8) | 20.3 (13.0–25.9) | 22.8 (16.0–34.0) | 28.8 (17.7–39.5) | 38.3 (20.3–51.2) |
| **Posterior Deltoid** | | | | | | |
| | Healthy | 2.2 (1.7–3.7) | 2.8 (2.0–4.3) | 3.8 (2.5–5.4) | 5.5 (3.5–7.1) | 7.0 (4.8–10.9) |
| | RC Tendinopathy | 4.1 (3.1–5.8) | 5.4 (3.4–8.3) | 7.2 (4.3–11.7) | 9.1 (5.8–13.7) | 10.1 (6.1–17.7) |
| | Asymptomatic RCT | 6.3 (2.7–9.21) | 9.12 (3.7–14.4) | 8.8 (5.3–16.9) | 12.4 (6.8–25.6) | 13.5 (8.0–38.4) |
| | Symptomatic RCT | 9.1 (5.7–18.1) | 13.0 (7.6–24.7) | 18.7 (10.4–25.7) | 22.3 (13.5–37.6) | 28.2 (18.8–43.9) |
| **Infraspinatus** | | | | | | |
| | Healthy | 6.5 (5.0–9.4) | 9.5 (6.8–12.2) | 11.8 (8.4–16.0) | 16.4 (11.0–20.4) | 20.6 (12.7–28.0) |
| | RC Tendinopathy | 8.2 (6.8–16.6) | 14.4 (8.2–20.9) | 16.9 (9.9–27.0) | 17.7 (12.2–27.8) | 25.7 (14.9–33.6) |
| | Asymptomatic RCT | 14.0 (8.1–20.0) | 17.8 (10.3–26.1) | 25.4 (13.4–37.2) | 29.5 (18.5–44.2) | 36.6 (21.2–54.5) |
| | Symptomatic RCT | 14.1 (9.9–24.3) | 20.3 (14.6–28.4) | 26.5 (18.2–36.4) | 33.8 (20.7–46.4) | 39.2 (31.4–59.3) |
| **Biceps Brachii** | | | | | | |
| | Healthy | 2.5 (1.6–3.1) | 3.4 (2.6–4.9) | 5.6 (4.0–10.9) | 7.3 (4.1–11.6) | 8.6 (5.2–16.8) |
| | RC Tendinopathy | 6.1 (3.2–9.8) | 7.3 (5.2–12.1) | 14.9 (7.5–20.8) | 14.4 (9.5–23.4) | 21.4 (11.3–34.6) |
| | Asymptomatic RCT | 6.0 (3.7–9.6) | 10.6 (6.4–16.6) | 15.3 (10.8–25.1) | 21.2 (15.1–32.7) | 24.2 (14.0–43.8) |
| | Symptomatic RCT | 9.5 (4.8–13.8) | 17.4 (6.5–25.4) | 21.4 (8.5–30.1) | 27.3 (9.5–45.0) | 25.8 (10.8–48.9) |

| | | Muscle activities (%MVC)<br>Median<br>(interquartile range) | | | | |
|---|---|---|---|---|---|---|
| | | *0kg* | *1kg* | *2kg* | *3kg* | *4kg* |
| **Latissimus Dorsi** | | | | | | |
| | Healthy | 3.2<br>(2.5–5.5) | 3.7<br>(2.4–5.8) | 3.8<br>(2.5–6.0) | 4.2<br>(2.5–6.5) | 4.6<br>(2.7–7.3) |
| | RC Tendinopathy | 8.2<br>(3.9–17.8) | 9.4<br>(4.7–18.0) | 8.5<br>(4.6–18.6) | 8.6<br>(4.5–18.5) | 8.7<br>(5.1–18.9) |
| | Asymptomatic RCT | 8.0<br>(5.8–17.1) | 8.2<br>(5.8–14.6) | 8.1<br>(6.5–20.4) | 9.7<br>(7.0–15.2) | 10.7<br>(7.2–22.0) |
| | Symptomatic RCT | 10.1<br>(7.0–16.4) | 12.2<br>(7.3–18.0) | 11.9<br>(8.1–20.4) | 13.3<br>(8.3–20.1) | 13.5<br>(9.4–22.5) |
| **Pectoralis Major** | | | | | | |
| | Healthy | 2.3<br>(1.5–3.7) | 3.1<br>(1.7–5.2) | 4.5<br>(2.5–7.2) | 5.4<br>(3.2–9.0) | 7.1<br>(4.1–11.0) |
| | RC Tendinopathy | 4.9<br>(3.3–9.3) | 6.7<br>(3.6–11.8) | 7.9<br>(4.5–13.7) | 8.9<br>(5.1–14.6) | 12.6<br>(7.6–16.1) |
| | Asymptomatic RCT | 4.6<br>(2.3–6.4) | 4.5<br>(2.7–6.7) | 6.8<br>(3.1–10.2) | 7.1<br>(3.8–13.0) | 9.5<br>(5.9–14.9) |
| | Symptomatic RCT | 8.8<br>(4.4–11.6) | 10.1<br>(6.8–14.1) | 13.2<br>(9.9–18.8) | 15.3<br>(7.9–20.4) | 16.4<br>(8.2–25.5) |
| **Upper Trapezius** | | | | | | |
| | Healthy | 8.4<br>(5.8–11.9) | 10.8<br>(7.9–16.6) | 12.6<br>(9.0–20.1) | 16.3<br>(11.5–25.6) | 21.2<br>(12.7–30.3) |
| | RC Tendinopathy | 13.6<br>(9.2–18.2) | 15.6<br>(12.2–24.4) | 19.4<br>(14.8–33.0) | 21.5<br>(17.1–36.6) | 25.1<br>(21.1–40.6) |
| | Asymptomatic RCT | 14.2<br>(10.5–18.6) | 18.6<br>(14.7–28.0) | 20.7<br>(15.4–32.6) | 24.0<br>(17.1–35.8) | 29.3<br>(21.4–54.0) |
| | Symptomatic RCT | 19.1<br>(12.0–24.4) | 21.8<br>(17.5–31.9) | 32.7<br>(18.7–36.2) | 33.1<br>(22.2–45.7) | 36.6<br>(27.8–61.4) |

RC, Rotator cuff; RCT, Rotator cuff tear; MVC, muscle voluntary contraction.
