# Supplementary material for: Load-induced increase in muscle activity during 30° abduction in patients with rotator cuff tears and control subjects
Source: J Orthop Traumatol. 2023 Aug 4;24:41. doi: 10.1186/s10195-023-00720-8 (PMC10403481; doi:10.1186/s10195-023-00720-8)
Supplement: Supplementary file 3 — Additional file 3: Table S3. P-values for the effects of load and shoulder types of the linear mixed models on the log-transformed muscle activities with change of reference. [file 10195_2023_720_MOESM3_ESM.pdf]

Table S3: P-values for the effects of load and shoulder types of the linear mixed models on the log-transformed muscle activities with change of reference.

**Predictors**	**Anterior Deltoid**	**Middle Deltoid**	**Posterior Deltoid**	**Infraspinatus**	**Biceps Brachii**	**Latissimus Dorsi**	**Pectoralis Major**	**Upper Trapezius**
(Intercept)								
Ref: Healthy	**<0.001**	**<0.001**	**<0.001**	**<0.001**	**<0.001**	**<0.001**	**<0.001**	**<0.001**
Ref: RC Tendinopathy	**<0.001**	**<0.001**	**<0.001**	**<0.001**	**<0.001**	**<0.001**	**<0.001**	**<0.001**
Ref: Asymptomatic RCT	**<0.001**	**<0.001**	**<0.001**	**<0.001**	**<0.001**	**<0.001**	**<0.001**	**<0.001**
Ref: Symptomatic RCT	**<0.001**	**<0.001**	**<0.001**	**<0.001**	**<0.001**	**<0.001**	**<0.001**	**<0.001**
Load								
Ref: Healthy	**<0.001**	**<0.001**	**<0.001**	**<0.001**	**<0.001**	**<0.001**	**<0.001**	**<0.001**
Ref: RC Tendinopathy	**<0.001**	**<0.001**	**<0.001**	**<0.001**	**<0.001**	**0.023**	**<0.001**	**<0.001**
Ref: Asymptomatic RCT	**<0.001**	**<0.001**	**<0.001**	**<0.001**	**<0.001**	**<0.001**	**<0.001**	**<0.001**
Ref: Symptomatic RCT	**<0.001**	**<0.001**	**<0.001**	**<0.001**	**<0.001**	**<0.001**	**<0.001**	**<0.001**
Healthy								
Ref: Healthy	-	-	-	-	-	-	-	-
Ref: RC Tendinopathy	**0.001**	**0.001**	**0.002**	**0.002**	**0.006**	**<0.001**	**<0.001**	**0.001**
Ref: Asymptomatic RCT	**<0.001**	**<0.001**	**<0.001**	**<0.001**	**<0.001**	**<0.001**	**0.031**	**<0.001**
Ref: Symptomatic RCT	**<0.001**	**<0.001**	**<0.001**	**<0.001**	**<0.001**	**<0.001**	**<0.001**	**<0.001**
RC Tendinopathy								
Ref: Healthy	**<0.001**	**0.001**	**0.002**	**0.002**	**0.006**	**<0.001**	**<0.001**	**0.001**
Ref: RC Tendinopathy	-	-	-	-	-	-	-	-
Ref: Asymptomatic RCT	**0.031**	0.277	0.222	0.160	0.429	0.764	0.107	0.356
Ref: Symptomatic RCT	**<0.001**	**0.001**	**<0.001**	**0.031**	0.056	0.548	0.125	0.133
Asymptomatic RCT								
Ref: Healthy	**<0.001**	**<0.001**	**<0.001**	**<0.001**	**<0.001**	**<0.001**	**0.031**	**<0.001**
Ref: RC Tendinopathy	**0.031**	0.277	0.222	0.160	0.429	0.764	0.107	0.356
Ref: Asymptomatic RCT	-	-	-	-	-	-	-	-
Ref: Symptomatic RCT	**0.022**	**0.015**	**0.004**	0.333	0.169	0.725	**0.001**	0.465
Symptomatic RCT								
Ref: Healthy	**<0.001**	**<0.001**	**<0.001**	**<0.001**	**<0.001**	**<0.001**	**<0.001**	**<0.001**
Ref: RC Tendinopathy	**<0.001**	**0.001**	**<0.001**	**0.031**	0.056	0.548	0.125	0.133
Ref: Asymptomatic RCT	**0.022**	**0.015**	**0.004**	0.333	0.169	0.725	**0.001**	0.465
Ref: Symptomatic RCT	-	-	-	-	-	-	-	-

Predictors		Anterior Deltoid	Middle Deltoid	Posterior Deltoid	Infraspinatus	Biceps Brachii	Latissimus Dorsi	Pectoralis Major	Upper Trapezius
Load x Healthy									
	Ref: Healthy	-	**-**	-	-	-	**-**	-	-
	Ref: RC Tendinopathy	**<0.001**	**0.011**	0.252	**<0.001**	0.985	**0.011**	0.135	0.068
	Ref: Asymptomatic RCT	**0.003**	0.600	0.266	0.447	0.060	0.171	0.139	0.027
	Ref: Symptomatic RCT	**0.006**	0.350	0.317	0.759	0.237	0.571	0.291	0.633
Load x RC Tendinopathy									
	Ref: Healthy	**<0.001**	**0.011**	0.252	**<0.001**	0.985	**0.011**	0.135	0.068
	Ref: RC Tendinopathy	**-**	-	-	**-**	-	-	-	-
	Ref: Asymptomatic RCT	0.374	**0.045**	**0.039**	**0.004**	0.112	**<0.001**	0.906	0.895
	Ref: Symptomatic RCT	0.497	0.162	0.059	**0.001**	0.308	**0.007**	0.723	0.246
Load x Asymptomatic RCT									
	Ref: Healthy	**0.003**	0.600	0.266	0.447	0.060	0.171	0.139	**0.027**
	Ref: RC Tendinopathy	0.374	**0.045**	**0.039**	**0.004**	0.112	**<0.001**	0.906	0.895
	Ref: Asymptomatic RCT	-	-	-	-	-	-	-	-
	Ref: Symptomatic RCT	0.891	0.645	0.979	0.346	**0.007**	0.536	0.795	0.162
Load x Symptomatic RCT									
	Ref: Healthy	**0.006**	0.350	0.317	0.759	0.237	0.571	0.291	0.633
	Ref: RC Tendinopathy	0.497	0.162	0.059	**0.001**	0.308	**0.007**	0.723	0.246
	Ref: Asymptomatic RCT	0.891	0.645	0.979	0.346	**0.007**	0.536	0.795	0.162
	Ref: Symptomatic RCT	-	-	-	-	-	-	-	-

Bold values indicate significant differences ($P < 0.05$). RC, Rotator cuff; RCT, Rotator cuff tear; Ref, Reference.
