# Supplementary material for: Load-induced increase in muscle activity during 30° abduction in patients with rotator cuff tears and control subjects
Source: J Orthop Traumatol. 2023 Aug 4;24:41. doi: 10.1186/s10195-023-00720-8 (PMC10403481; doi:10.1186/s10195-023-00720-8)
Supplement: Supplementary file 4 — Additional file 4: Table S3. P-values of the post hoc tests of the log-transformed muscle activities for the shoulder effect. [file 10195_2023_720_MOESM4_ESM.pdf]

Table S4: P-values of the post hoc tests of the log-transformed muscle activities for the shoulder effect.

		Healthy vs. RC Tendinopathy	*Healthy vs. Asymptomatic RCT*	*Healthy vs. Symptomatic RCT*	*RC Tendinopathy vs. Asymptomatic RCT*	*RC Tendinopathy vs. Symptomatic RCT*	*Asymptomatic RCT vs. Symptomatic RCT*
**Anterior Deltoid**							
	0 kg	**0.005**	**<0.001**	**<0.001**	0.139	**<0.001**	0.103
	1 kg	**0.016**	**<0.001**	**<0.001**	0.099	**<0.001**	0.096
	2 kg	0.051	**<0.001**	**<0.001**	0.074	**<0.001**	0.096
	3 kg	0.146	**<0.001**	**<0.001**	0.059	**<0.001**	0.104
	4 kg	0.344	**<0.001**	**<0.001**	0.051	**<0.001**	0.120
**Middle Deltoid**							
	0 kg	**0.008**	**<0.001**	**<0.001**	0.696	**0.009**	0.073
	1 kg	**0.015**	**<0.001**	**<0.001**	0.559	**0.005**	0.075
	2 kg	**0.030**	**<0.001**	**<0.001**	0.427	**0.003**	0.082
	3 kg	0.060	**<0.001**	**<0.001**	0.318	**0.002**	0.094
	4 kg	0.120	**<0.001**	**<0.001**	0.235	**0.001**	0.113
**Posterior Deltoid**							
	0 kg	**0.015**	**<0.001**	**<0.001**	0.614	**0.002**	**0.025**
	1 kg	**0.018**	**<0.001**	**<0.001**	0.464	**<0.001**	**0.022**
	2 kg	**0.024**	**<0.001**	**<0.001**	0.334	**<0.001**	**0.021**
	3 kg	**0.035**	**<0.001**	**<0.001**	0.234	**<0.001**	**0.022**
	4 kg	0.054	**<0.001**	**<0.001**	0.164	**<0.001**	**0.024**
**Infraspinatus**							
	0 kg	**0.011**	**<0.001**	**<0.001**	0.498	0.141	0.768
	1 kg	**0.031**	**<0.001**	**<0.001**	0.305	0.065	0.696
	2 kg	0.081	**<0.001**	**<0.001**	0.168	**0.018**	0.627
	3 kg	0.195	**<0.001**	**<0.001**	0.087	**0.006**	0.566
	4 kg	0.399	**<0.001**	**<0.001**	**0.045**	**0.002**	0.516
**Biceps Brachii**							
	0 kg	**0.031**	**<0.001**	**<0.001**	0.858	0.227	0.517
	1 kg	**0.026**	**<0.001**	**<0.001**	0.739	0.267	0.725
	2 kg	**0.025**	**<0.001**	**<0.001**	0.605	0.328	0.904
	3 kg	**0.027**	**<0.001**	**<0.001**	0.482	0.414	0.990
	4 kg	**0.033**	**<0.001**	**<0.001**	0.384	0.519	0.999

	Healthy vs. RC Tendinopathy	*Healthy vs. Asymptomatic RCT*	*Healthy vs. Symptomatic RCT*	*RC Tendinopathy vs. Asymptomatic RCT*	*RC Tendinopathy vs. Symptomatic RCT*	*Asymptomatic RCT vs. Symptomatic RCT*
**Latissimus Dorsi**						
0 kg	**<0.001**	**<0.001**	**<0.001**	0.991	0.931	0.985
1 kg	**<0.001**	**<0.001**	**<0.001**	0.956	0.871	0.989
2 kg	**<0.001**	**<0.001**	**<0.001**	0.889	0.793	0.992
3 kg	**<0.001**	**<0.001**	**<0.001**	0.789	0.703	0.995
4 kg	**0.002**	**<0.001**	**<0.001**	0.670	0.609	0.997
**Pectoralis Major**						
0 kg	**0.002**	0.140	**<0.001**	0.373	0.418	**0.008**
1 kg	**0.003**	0.188	**<0.001**	0.358	0.366	**0.006**
2 kg	**0.005**	0.267	**<0.001**	0.359	0.331	**0.005**
3 kg	**0.010**	0.382	**<0.001**	0.377	0.315	**0.005**
4 kg	**0.023**	0.530	**<0.001**	0.411	0.315	**0.005**
**Upper Trapezius**						
0 kg	**0.004**	**<0.001**	**<0.001**	0.792	0.438	0.884
1 kg	**0.006**	**<0.001**	**<0.001**	0.791	0.350	0.805
2 kg	**0.011**	**<0.001**	**<0.001**	0.796	0.280	0.714
3 kg	**0.020**	**<0.001**	**<0.001**	0.806	0.228	0.623
4 kg	**0.039**	**<0.001**	**<0.001**	0.822	0.192	0.540

Bold values indicate significant differences ($P < 0.05$). RC, Rotator cuff; RCT, Rotator cuff tear.
