# Supplementary material for: Load-induced increase in muscle activity during 30° abduction in patients with rotator cuff tears and control subjects
Source: J Orthop Traumatol. 2023 Aug 4;24:41. doi: 10.1186/s10195-023-00720-8 (PMC10403481; doi:10.1186/s10195-023-00720-8)

Figure S1: Absolute difference in the muscle activity of maximum voluntary contraction between sides for each group. Note: participants were sorted from smallest to largest difference between sides within each group. MVC, muscle voluntary contraction; mV, millivolts.

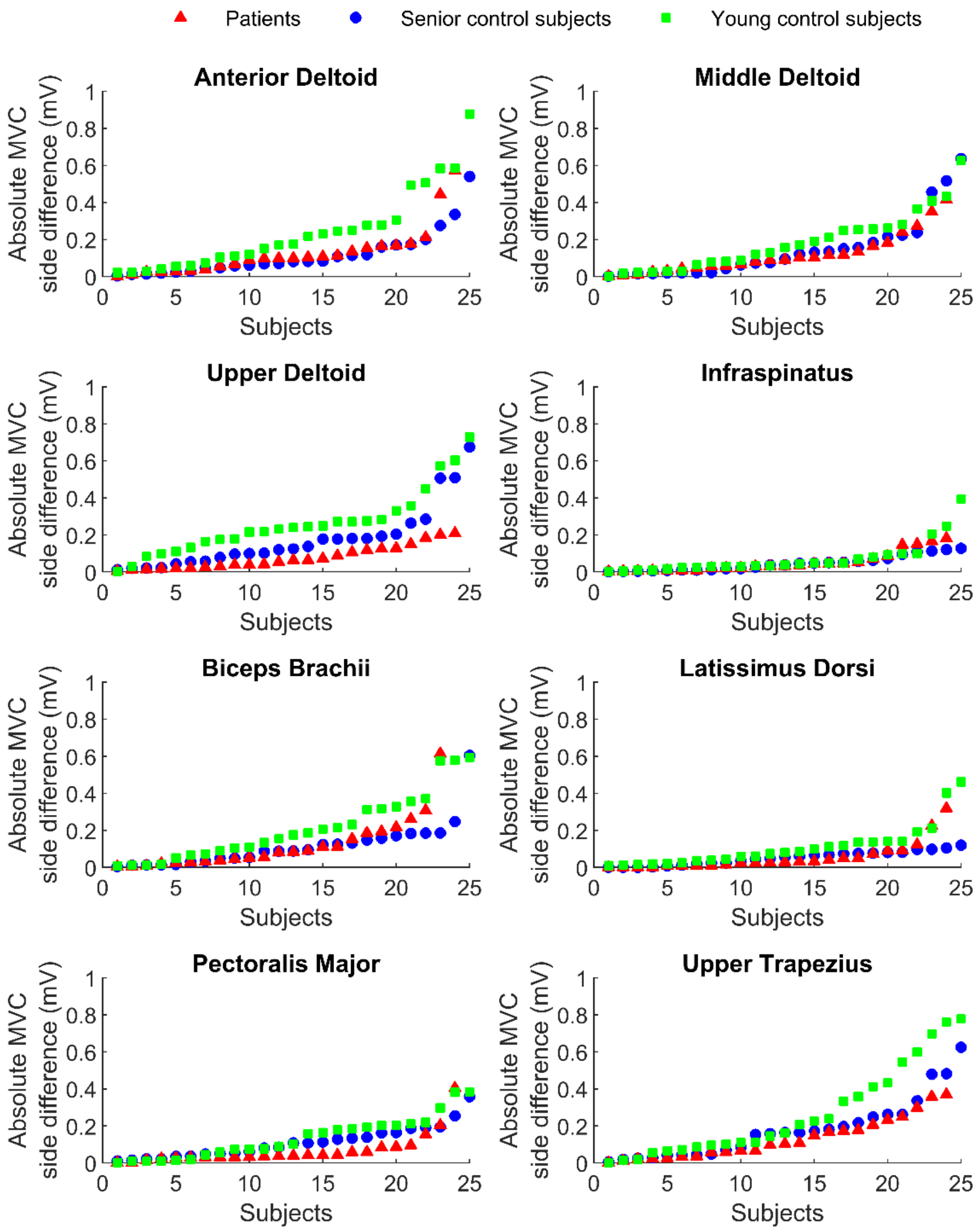

Supplement: Supplementary file 5 — Additional file 5: Figure S1. Absolute difference in the muscle activity of maximum voluntary contraction between sides for each group. [file 10195_2023_720_MOESM5_ESM.pdf]
